# Supplementary material for: MetaRibo-Seq measures translation in microbiomes
Source: Nat Commun. 2020 Jun 29;11:3268. doi: 10.1038/s41467-020-17081-z (PMC7324362; doi:10.1038/s41467-020-17081-z)
Supplement: Supplementary file 10 — Supplementary Data 7 [file 41467_2020_17081_MOESM10_ESM.zip › File2/Confidence_VeryHigh_Taxonomy/5988_out.krona.html]

Javascript must be enabled to view this page.

members
magnitude
magnitudeUnassigned
count
unassigned
taxon
rank

5988\_out

13

2

SRS015663\_contig\_number\_contig-100\_8029.129177SRS143070\_contig\_number\_contig-100\_41610.41610

2
11
superkingdom

1239
phylum
11

186801
11
class

11
order
186802

541000
family
11

11
genus
216851

2302955
1

SRS143895\_contig\_number\_36890
species

10
species

SRS012849\_contig\_number\_24483SRS013951\_contig\_number\_contig-100\_287.225209SRS015431\_contig\_number\_88490SRS018836\_contig\_number\_26013SRS019068\_contig\_number\_99512SRS019161\_contig\_number\_9863SRS019496\_contig\_number\_contig-100\_6.112896SRS076756\_contig\_number\_20960SRS104693\_contig\_number\_13184SRS148196\_contig\_number\_40161
853
